# Optimized IoT protocol stack for seamless smart home communication using Random Forest-based interoperability analysis

**DOI:** 10.1038/s41598-025-23948-2

**Published:** 2025-11-17

**Authors:** A. Sriram, D. Manikandan, R. Venketesan, Krishnaraj Ramaswamy

**Affiliations:** 1 Department of Electronics and Communication Engineering, SRM TRP Engineering College, Tiruchirappalli, Tamilnadu India; 2 Department of Mechanical Engineering, SRM TRP Engineering College, Tiruchirappalli, Tamilnadu India; 3 Department of Mechanical Engineering, College of Engineering and Technology, Dambi Dollo University, Dambi Dollo, Ethiopia; 4 Center for Global Health Research, Saveetha Institute of Medical and Technical Sciences, Saveetha University, Chennai, Tamilnadu India

**Keywords:** IoT, Smart home, Protocol stack, Interoperability, Random forest, Seamless communication, Machine learning, Adaptive interface, Device compatibility, Intelligent system, Engineering, Mathematics and computing

## Abstract

This paper introduces an optimised and harmonised Internet of Things (IoT) protocol stack aimed at enabling seamless device communication in smart home settings, utilising a Random Forest-based interworking analysis framework. Smart homes usually have a lot of different devices that use different communication protocols, which makes it very hard for them to work together without problems. To solve these problems, we suggest a self-adaptive, machine learning–based networking protocol stack that can quickly check the state of the network and improve how protocols work together. The Random Forest algorithm is used to find hidden connectivity patterns, predict the best ways for devices to talk to each other, and sort device interactions based on both past and present data. The system architecture includes a context-aware protocol manager, a learning-driven communication controller, and adaptive interface mapper layers that all work together to make sure that data is exchanged smoothly and coherently. Tests show that the system consistently has a communication success rate of over 85% and a compatibility score of over 0.7 for 70% of the time it is in use. In 70% of cases, latency is kept under 150 milliseconds. The solution greatly improves the interconnectivity, energy efficiency, and overall user experience of smart home devices by adding multiple intelligent layers. The built-in AI framework also saves up to 30% on energy costs while making the home safer and more comfortable. This improves the capabilities of smart home automation and user interaction.

## Introduction

The The Internet of Things (IoT) has grown so quickly that it has turned regular homes into smart environments where many devices talk to each other and work together to make life easier, safer, and more energy-efficient^1^. But smart homes have a lot of different devices and communication protocols, which makes it hard to make them all work together smoothly^2^. Devices from different companies often use proprietary or incompatible standards, which makes systems less efficient and communication less reliable, which makes smart home automation less effective^3^. To solve these problems, we need to make systems that can dynamically manage and optimise protocol interactions so that different devices can communicate with each other reliably^4^.

Machine learning (ML) is a popular tool for IoT environments because it lets systems learn from data, find patterns, and make decisions that change over time without having to be explicitly programmed^5^. Supervised learning algorithms, like Random Forest, have shown to be very good at classification and prediction tasks. This makes them a good choice for solving interoperability problems in IoT networks^6^. An IoT protocol stack can be made to smartly find communication errors and improve how devices talk to each other by using both historical and real-time data^7^. Adding machine learning to protocol management has a lot of benefits. For example, it meets the growing technological needs of IoT systems and makes smart home networks work better^8^. These kinds of systems can change settings on their own based on device settings, network conditions, and user preferences. This makes sure that service is always available and improves the user experience^9^.

For an optimised ML-based IoT protocol stack makes smart homes truly smart, where devices talk to each other without any problems and automation works to its full potential^10^.

Even with these improvements, most studies still focus on middleware-based solutions, protocol gateways, or isolated ML techniques, which don’t make a full, adaptable protocol stack. Moreover, there are only a few methods that integrate machine learning-based interoperability analysis with real-time monitoring and adaptive protocol mapping. This paper proposes an optimised IoT protocol stack that incorporates Random Forest-based interoperability analysis, a context-aware protocol manager, and an adaptive interface mapper within a hybrid edge-cloud framework to fill this gap. This work is new because it combines different types of intelligence, such as classification, similarity-based clustering, and entropy-based uncertainty detection, into a single architecture that makes smart home devices work better together, reduces latency, and increases reliability.

## Literature survey

In Researchers have looked into many ways to make IoT communication and connectivity better as they work on smart home technologies for the 21 st century. One study put forward a lightweight protocol adaptation framework to connect protocols that don’t work with each other, making sure that data can be exchanged consistently across platforms^11^. Another study showed a middleware-based architecture that used semantic interoperability to let devices that use different standards talk to each other^12^. Researchers also came up with a dynamic protocol selection mechanism that picks the best communication protocol for a given situation based on what the devices can do and what they know about the situation^13^.

Another important contribution was the development of a multi-protocol gateway solution that can support multiple IoT standards at the same time. This speeds up communication delays and the overall response time of the system^14^. Machine learning methods have also been used to get around protocol and communication problems in smart homes that use the Internet of Things. One study, for example, used decision tree classifiers to look at how devices worked and guess which protocols would work best for smooth communication^15^. A deep learning-based framework was also suggested to predict changes that need to be made to communication patterns in smart home settings. This is a promising way to make different systems work together better^16^.

Subsequent research incorporated reinforcement learning to autonomously determine the most efficient communication pathways and optimise essential protocol parameters. This method was able to adapt to changes in the environment and find the best communication paths in real time^17^. Clustering algorithms have also been shown to be good for grouping devices on a network, which makes protocol mapping more efficient and personalised^18^. Recent studies have also focused on combining machine learning with edge computing to make Industrial IoT (IIoT) and real-time decision-making possible in smart homes with less lag time^19^.

Anthony^20^ recently suggested decentralised AIoT-based intelligence to facilitate sustainable energy prosumption, highlighting the importance of citizen-centric interoperability in smart communities. Hazman et al.^21^ conducted an extensive review of anomaly detection techniques for the security of IoT systems, highlighting that resilient interoperability must include security-conscious designs. Machele et al.^22^ conducted a survey on interconnected smart transactive microgrids, emphasising the significance of communication interoperability for energy trading and optimisation. Mamun et al.^23^ also showed an AI-driven smart reception framework that combined multimodal recognition. This shows that there is a growing need for real-time adaptability in IoT-enabled environments.

Recent research has concentrated on intrusion detection and system dependability. Manivannan^24^ examined machine learning-based intrusion detection systems for the Internet of Things (IoT), while Nandanwar and Katarya^25^ presented a deep learning-enabled intrusion detection system (IDS) for industrial IoT, both highlighting the essential function of adaptive communication layers in heterogeneous networks. Shah et al.^26^ also looked into AI-enhanced collaborative robotics, focussing on human-centered automation where safe and smooth interoperability is just as important.

**Fig. 1:**
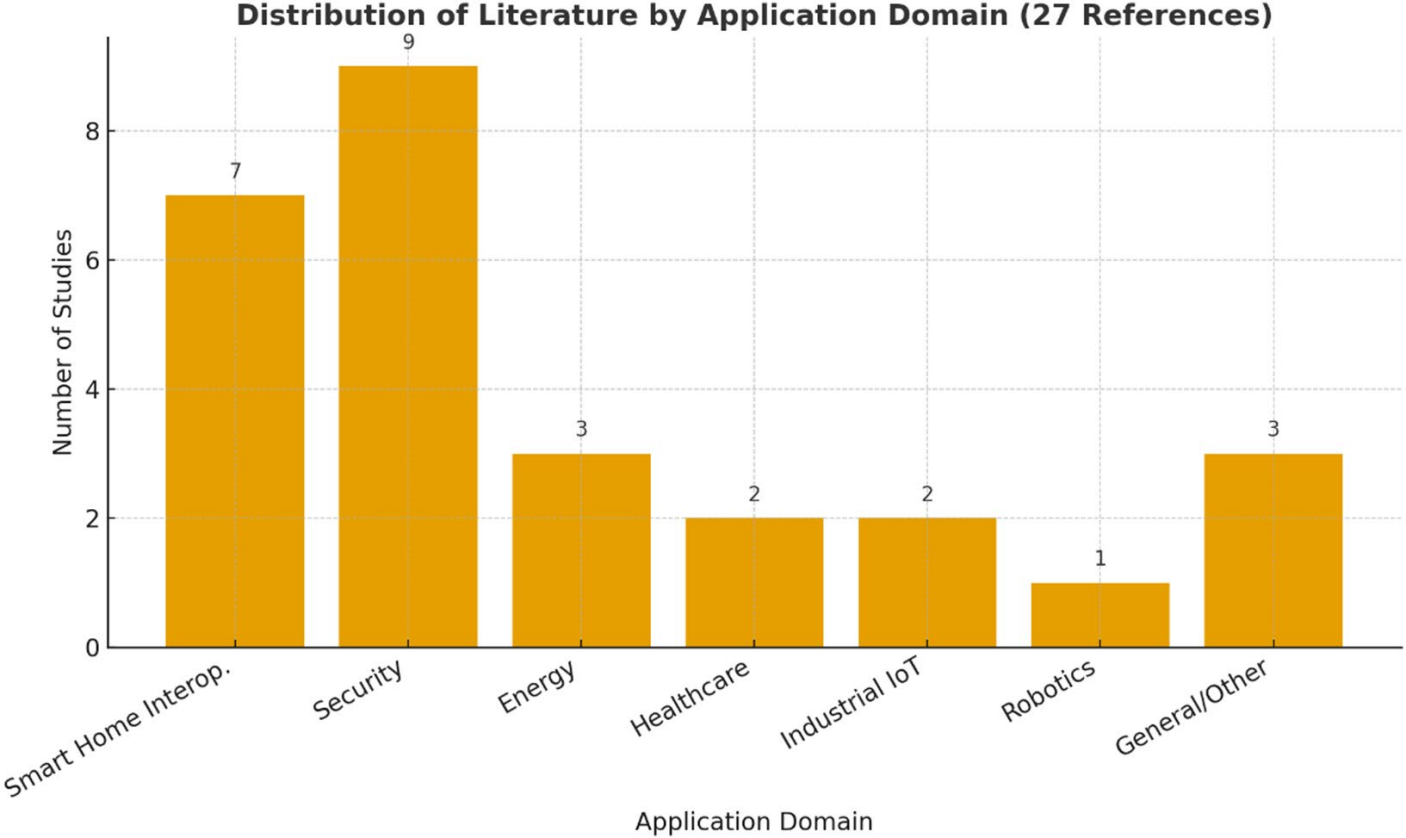
Distribution across IoT application domains from the reviewed papers.

Figure 1 shows how 27 reviewed references are grouped by different IoT application areas. It is evident that security and smart home interoperability are the predominant research areas, suggesting that the majority of efforts are concentrated on anomaly detection, intrusion prevention, and device communication issues within heterogeneous IoT networks. There are only three studies on energy applications, two on healthcare systems, and two on industrial IoT. Only one study looks at robotics. The other three studies are either general surveys or minor contributions. This distribution shows that even though interoperability and security have gotten a lot of attention, there still aren’t any complete IoT protocol stack solutions that use machine learning to make communication in smart homes easier. The works that are already out there are either focused on a specific field (like energy, healthcare, or on individual parts, like gateways or intrusion detection. Conversely, the current study fills this void by presenting an enhanced IoT protocol stack featuring Random Forest-based interoperability analysis, adaptive mapping, and a hybrid edge-cloud architecture, thereby providing a more cohesive and intelligent framework for smart home settings.

**Table 1:** Comparative overview of common smart home IoT communication Protocols.

Protocol	Range	Data Rate	Power Consumption	Security	Typical Use Cases
ZigBee	10–100 m	Up to 250 kbps	Low	AES-128 encryption	Home automation, sensors
Z-Wave	30–100 m	Up to 100 kbps	Very low	AES-128 encryption	Security systems, locks
Matter	10–30 m	Varies	Low	End-to-end encryption	Cross-brand interoperability
Wi-Fi	30–100 m	Up to 1 Gbps	Moderate to high	WPA3 encryption	High bandwidth devices
Thread	10–100 m	Up to 250 kbps	Low	AES-128 encryption	Smart lighting, sensors
Bluetooth LE	10–100 m	Up to 2 Mbps	Very low	AES-128 encryption	Wearables, remote controls
LoRaWAN	Up to 15 km	Up to 50 kbps	Very low	AES-128 encryption	Long-range IoT applications
KNX	10–100 m	Varies	Low	End-to-end encryption	Building automation, HVAC

Table 1 presents a comparative overview of prevalent smart home IoT communication protocols. Each protocol differs significantly in operational range, data throughput, power consumption, security mechanisms, and typical application scenarios. Understanding these distinctions provides insight into the heterogeneity and complexity of smart home networks, underscoring the need for an adaptive, machine learning-based protocol stack to facilitate inter-protocol communication and enhance network reliability.

For the ensemble methods like Random Forest have been shown to work very well for classifying communication control and protocol analysis tasks^27^. Although previous research has examined middleware solutions, deep learning, and reinforcement learning, there is a scarcity of studies proposing a comprehensive IoT protocol stack that amalgamates Random Forest–based interoperability analysis with adaptive interface mapping and entropy-driven monitoring within a hybrid edge cloud architecture. This research gap drives the current study, which seeks to provide a comprehensive and sophisticated solution for seamless communication within smart homes.

## Proposed system

The proposed system employs a structured processing approach to ensure effective interoperability of IoT protocols in smart home communication. At the outset, communication data is sourced from heterogeneous smart home devices operating under different protocols. To prepare this raw data for analysis, pre-processing is performed to reduce noise and standardize the format, thereby facilitating accurate decision-making. A key innovation of the system is the use of the Random Forest algorithm, which serves as the analytical engine to identify patterns, classify protocol-related device behaviors, and detect interactions, as illustrated in Figs. 1a The proposed IoT protocol stack operates on a hybrid edge–cloud architecture to ensure low-latency communication and scalable analytics for smart home environments. At the Device Layer, heterogeneous smart appliances and sensors communicate via ZigBee, Wi-Fi, and Bluetooth Low Energy (BLE) protocols. These data streams are processed at the Edge Layer, where the Protocol Manager dynamically selects and adapts communication protocols, and the Interface Mapper performs real-time command and data translation between incompatible devices. A Local Data Store maintains short-term interaction logs and performance metrics to support immediate decision-making and reduce reliance on external connectivity. For more computationally intensive tasks, such as model retraining and long-term analytics, the Cloud Layer hosts the Random Forest engine and advanced analytics modules. A User Interface accessible via a mobile app or web portal allows users to configure devices, monitor performance, and view interoperability insights. This architecture balances local processing for fast responses with optional cloud support for scalability and advanced intelligence, making the system both practical and adaptable for diverse smart home deployments. The ensemble nature of the Random Forest algorithm ensures that both historical and real-time data streams are leveraged to generate robust and adaptive solutions.

In addition, the system incorporates a context-aware protocol manager capable of dynamically adjusting protocol layers based on device operating conditions and communication requirements. An adaptive interface mapper is also provided to enable seamless cross-translation of commands and data formats between devices. These coordinated components work together to maintain high levels of communication interoperability, minimize communication errors, and support intelligent, autonomous interactions among household appliances.

**Fig. 2:**
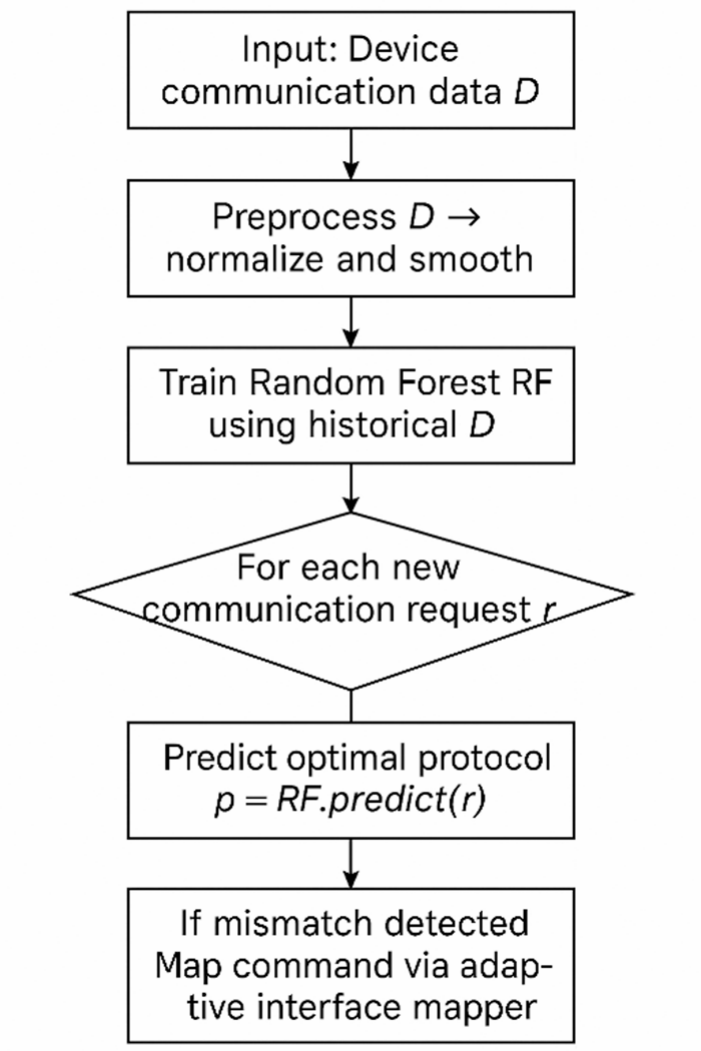
Flow of overall system efficiency and significance.

Collectively, these features shown in the Fig. 2, how it enhances overall system efficiency and significantly improves the smart home user experience. The final element of the system is a context-awareness module, which continually monitors network conditions to support proactive adjustments and ensure reliable performance. There are three main layers in the engineering design of the IoT protocol optimization system: the device layer, the edge layer, and the cloud layer. The edge layer works on smart home hubs or gateways that have embedded Linux or real-time operating systems like Yocto or Free RTOS. The cloud layer uses scalable cloud platforms like AWS or Azure and containerized microservices to make deployment more flexible. The device layer is made up of IoT devices that use ZigBee, BLE, and Wi-Fi modules’ native firmware and protocol stacks. In terms of hardware, edge devices usually use ARM Cortex-A series processors or something similar, and they have 16GB of RAM to run lightweight machine learning inference engines. Cloud servers have powerful CPUs with multiple cores and at least 32GB of RAM that are used to train the Random Forest models and do analytics. The IoT devices are limited in their own right, usually having microcontrollers that work in the MHz range and only a few hundred kilobytes of RAM. RESTful APIs or MQTT messaging make it easier for layers to talk to each other. On the other hand, lightweight protocols like ZeroMQ are used for local inter-process communication between parts like the Protocol Manager and Interface Mapper.

**Fig. 3:**
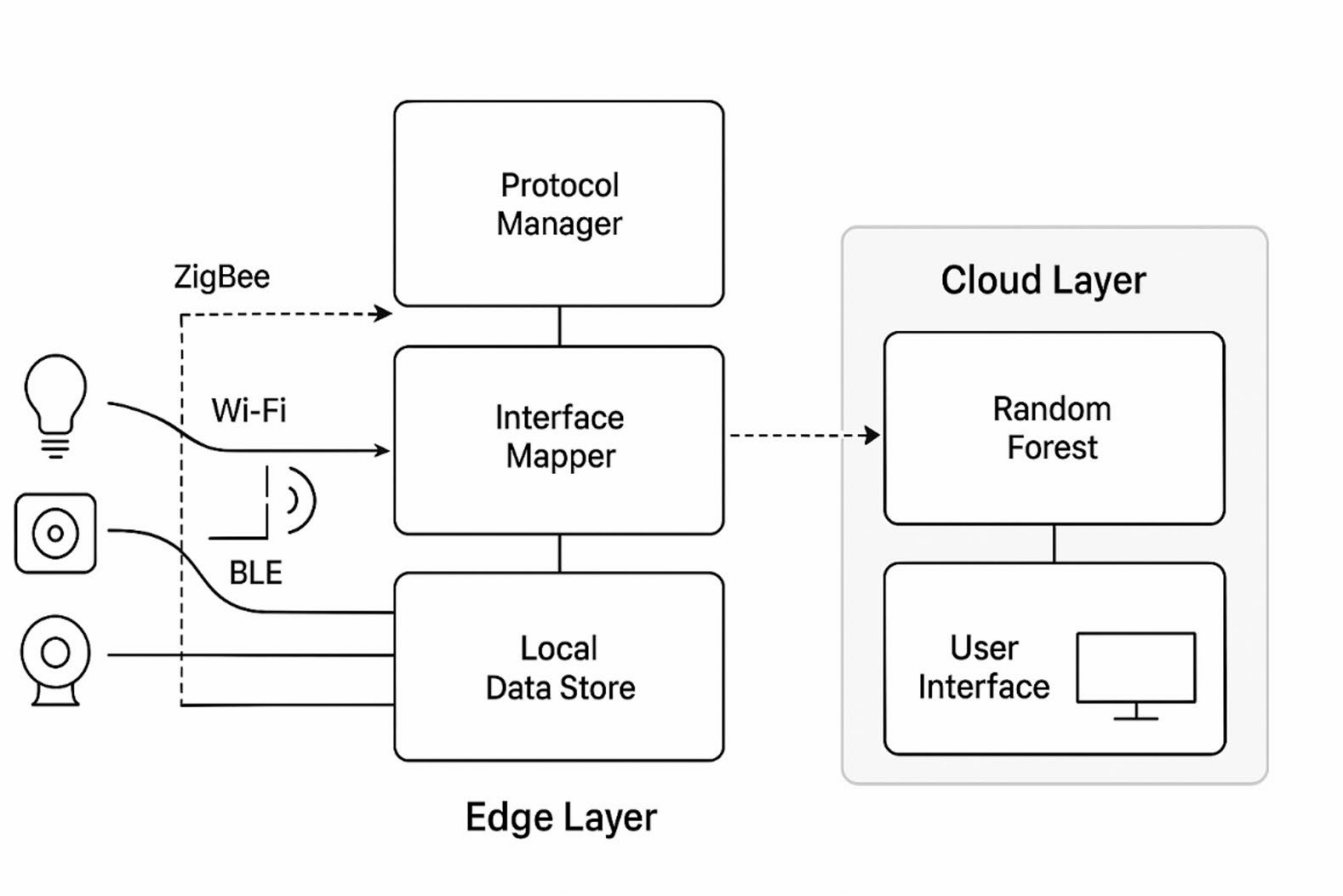
Flow of Proposed system.

Network interfaces like WLAN, ZigBee coordinators, and BLE controllers have low-level driver APIs that let them intercept and process raw packet data shown in the Fig. 3. Transport layer protocols like TLS or DTLS protect data exchanges. OAuth or token-based authentication schemes control who can access cloud services. Over-the-air methods send updates and model retraining from the cloud to edge devices. The protocol adaptation logic is written in fast, low-latency languages like C, C++, or Rust to improve performance. Data structures that use less memory and controls for concurrency keep overhead low on devices with limited resources. Local caching and batching policies also cut down on network load and save power. This practical engineering stack carefully balances real-world resource limits with advanced machine learning-driven protocol management. It gives us a way to put the ideas in this paper into action. The proposed approach takes the form of a step-by-step process in which the machine learning methods are utilized for the purpose of optimizing IoT protocol interoperability in smart homes with the help of a Random Forest model. At first, the system is designed to acquire communication data from the involved smart devices that are stored in the matrix as in (1).

Let x be the input feature vector for an IoT device communication instance:

1 $$\:x=\left[{f}_{1},{f}_{2},{f}_{3},\dots\:,{f}_{6}\right]$$

Where each feature $\:fi$ represents specific measurable attributes such as:

$\:{f}_{1}$ : Packet Size (bytes).

$\:{f}_{2}$ : Received Signal Strength Indicator (RSSI).

$\:{f}_{3\:}$ : Time of Day (encoded as numeric).

$\:{f}_{4\:}$ : Device Type (categorical, one-hot encoded).

$\:{f}_{5\:}$ : Protocol Type (categorical, one-hot encoded).

$$\:{f}_{6\:}$$ : Latency (milliseconds).

Context-Aware Protocol Manager’s decision function:

2 $$\:W\left(x\right)=\sum\:_{j=1}^{n}\:{a}_{j}{f}_{j}\left(x\right)$$

Where $\:{a}_{j}$ are learned weights reflecting the importance of each feature in the decision.

Random Forest prediction function aggregates the decisions of multiple trees $\:{T}_{m}$:

3 $$\:RF\left(x\right)=\text{m}\text{a}\text{j}\text{o}\text{r}\text{i}\text{t}\text{y}\left\{{T}_{1}\right(x),{T}_{2}(x),\dots\:,{T}_{M}(x\left)\right\}$$

Each decision tree $\:{T}_{m}$ applies splits on selected features $\:{f}_{j}$ to reach a classification or regression output.

Interface Mapping for protocol adaptation:

4 $$\:y=\mathcal{M}(x,{P}_{i},{P}_{j})$$

Where $\:x$ is the input message payload, and $\:{P}_{i}$, $\:{P}_{j}$ are protocols for incoming and outgoing communication. The mapping function $\:\mathcal{M}$ transforms the message data from protocol $\:{P}_{i}$ format to $\:{P}_{j}$.

Minimize classification error over dataset

5 $$\:D=\left\{\right({x}^{\left(i\right)},{y}^{\left(i\right)}){\}}_{i=1}^{N}$$

6 $$\:\underset{\left\{{T}_{m}\right\}}{\text{m}\text{i}\text{n}}\:\frac{1}{N}\sum\:_{i=1}^{N}\:\mathbb{I}\left(RF\right({x}^{\left(i\right)})\ne\:{y}^{\left(i\right)})$$

Where $\:\mathbb{I}$ is an indicator function marking misclassifications.

7 $$\:X=\left\{xmn\right\}.\:$$

where xmn is a variable signifies that the exchange of data is done between device m and device n in a period of time. Min-max scaling (8) is used for normalization which not only eliminates scale bias but also gives the data uniform treatment.

8 $$\:Xmn^{\prime\:}=xmn-Xmin/Xmax-Xmin.\:$$

In this case, Xmin and Xmax refer to the lowest and highest values of the dataset correspondingly, and Xmn′ represents the normalized data value whose scale is the same as that of interval 0 to 1. Such a transformation permits the model to deal with data unaffected by outlier values. Afterwards, the major part of the Random Forest model is applied to select N number of decision trees based on the given dataset using the bootstrap sampling procedure while the core of the Random Forest model is compiled by combining multiple output-based decision Tree. This averaging method serves a smoothing effect on the data follows where the device mm output is the mean value of the data points belonging to the device-m and its neighbors as shown in (9).:

9 $$\:Sm=1/k\sum\:ixm,i,(i=1\:to\:k).\:$$

The application at first step in the reduction process smooths the data: it also diminishes the fluctuation and enhances the signal reliability, i.e., the system margin of error is reduced before the training starts. The central layer of the framework is represented by a Random Forest classification model that is composed of N decision trees, each of them trained on one bootstrapped sample of the dataset. The probability of feature selection at each node split, is determined via the formula (10):

10 $$\:P\left(fj\right)=1/F.\:$$

where F is the total number of features available, and fj stands for the jth feature. A consistent selection ensures fair and unbiased resource allocation to all features. The classification outcome of the i-th tree hi (·) is derived using the collected data z. The final prediction of the Random Forest H (·) is carried out by the majority decision rule among all the trees as defined in (11):

11 $$\:H\left(z\right)=mode\left(h1\right(z),h2(z),\dots\:,hN(z\left)\right).$$

This ensemble approach improves prediction stability and accuracy by aggregating multiple weak learners. The confidence score C(z) of the prediction is calculated as the fraction of trees agreeing on the predicted classy in (12):

12 $$\:C\left(z\right)=1/N\sum\:il\left(hi\right(z)=y),(i=1\:to\:N)$$

where l is the indicator function returning 1 if true and 0 otherwise. Higher confidence values signify stronger consensus among trees. To facilitate protocol compatibility, the system computes similarity between devices’ communication profiles using cosine similarity by (13):

13 $$\:Sim(a,b)=a\cdot\:b/\parallel\:a\parallel\:\parallel\:b\parallel\:.$$

where vectors a and b denotes feature representations of two devices, ⋅ tells about the dot product, and ∥⋅∥ be Euclidean norm. This similarity parameter helps group devices with compatible communication patterns. The Clustering is formalized using a clustering approach, where the centroid of cluster cc at iteration t will be updated by (14):

14 $$\:\mu\:c\left(t\right)=1/\mid\:Sc\mid\:\sum\:x\in\:Scx.$$

Here, Sc is the set of data points assigned to cluster c, and µc(t) is the-cluster centroid that will represent the group’s communication characteristics. The context-aware protocol manager selects the communication paths by solving the problem of the maximum of the sum of the products of the features by (15):

15 $$\:W\left(x\right)=\sum\:j\alpha\:jfj\left(x\right),(j=1\:to\:F).\:$$

The feature functions fj(x) represents the features of the communication while αj are learned weights that tell how important they are. This decision making is weighted in a way that the protocol stack can smoothly handle the present network conditions and device contexts. The communication behavior’s uncertainty is calculated by the Shannon entropy in (16) :

16 $$\:H\left(p\right)=-\sum\:ipilogpi,(i=1\:to\:M).\:$$

where p={p1, p2,…,pM} is the probability distribution of communication states. The higher entropy values are initially less predictable, thus they denote a need for the system to react and perform some kind of adaptive effort. To solve the problem of transformation of data between different devices, the interface mapper changes the data according to the learned linear transformation in (17):

17 $$\:Y=MX.$$

Here is where X is the input data vector, M is the transformation matrix, and Y is the output suitable for the target device, which is coded for the rules of the protocol. The reconstruction error by (18) formula is the way for the optimal problem of the transformation matrix M.:

18 $$\:E=\parallel\:Y-Y*\parallel\:$$

where Y∗ is the ideal mapped output, and ∥⋅∥2 denotes the Euclidean norm, ensuring accurate protocol conversion. Continuous monitoring is performed by calculating the interaction success score Rmn over T time intervals by Eq. 19:

19 $$\:Rmn=\sum\:t\delta\:mn\left(t\right),(t=1\:to\:T).$$

where δmn(t) is a binary indicator of successful communication between devices mm and n at time t. These scores inform protocol adjustments to maintain high reliability. The overall protocol stack performance P is evaluated as the average success rate across all device pairs in (20):

20 $$\:P=1/M\times\:N\sum\:m\sum\:nsmn\:,(m=1\:to\:M),(n=1\:to\:N).$$

where smn is the success rate for the device pair (m, n). By iteratively applying these mathematical methods, the proposed system intelligently analyzes, adapts, and optimizes communication protocols, enabling seamless interoperability and robust smart home device communication.

**Fig. 4:**
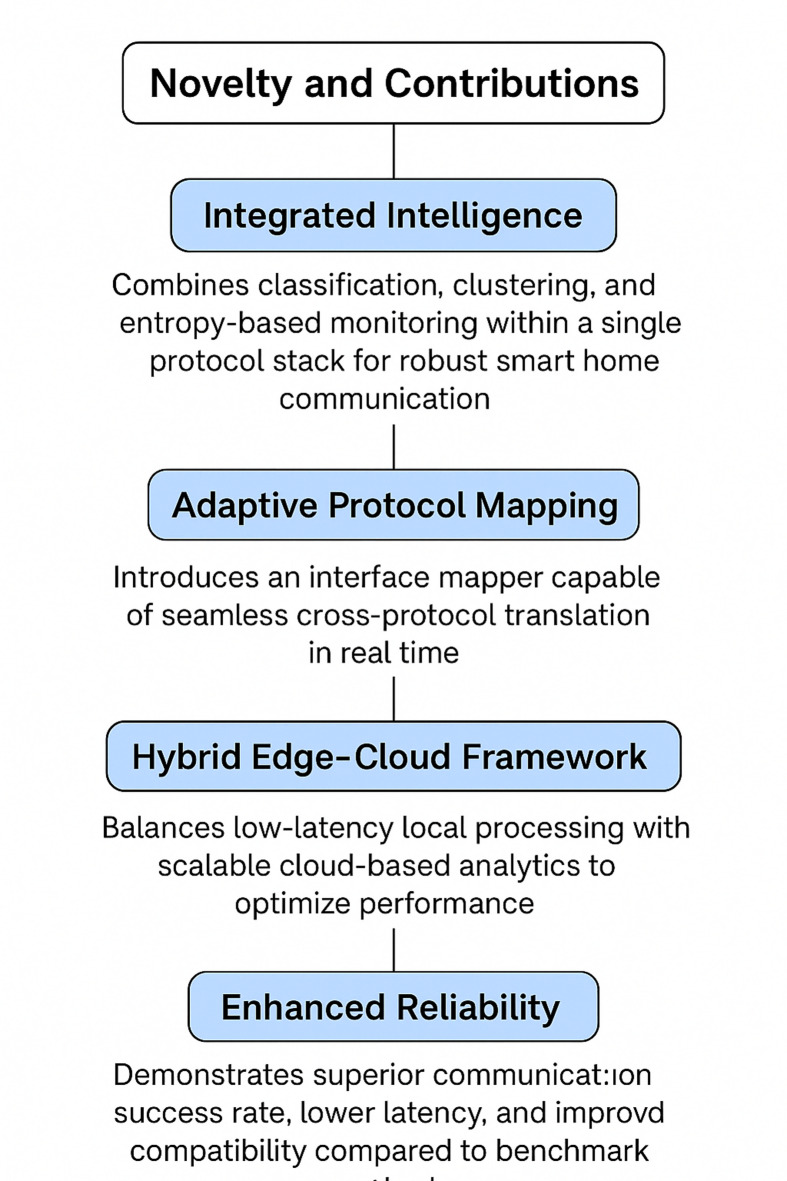
IoT Innovations and Contributions Flowchart.

This work uniquely combines Random Forest-based interoperability analysis, entropy-driven uncertainty detection, adaptive interface mapping, and a hybrid edge-cloud architecture into a single IoT protocol stack. This is different from existing middleware or isolated machine learning approaches shown the following Fig. 4.

This combination lets you change protocols in real time and group devices together in a way that previous methods couldn’t do at the same time. A context-aware protocol manager lets you proactively reconfigure your network when conditions change, which makes it more stable and adaptable. The main things this study adds are integrated Intelligence: This protocol stack brings together classification, clustering, and entropy-based monitoring to make smart home communication more reliable. Adaptive Protocol Mapping, adds an interface mapper that can translate between protocols in real time without any problems. The Hybrid Edge–Cloud Framework finds the best balance between fast local processing and cloud-based analytics that can grow to meet your needs. Better Reliability may Shows a higher success rate for communication, lower latency, and better compatibility than standard methods.

## Results and discussion

The optimized IoT protocol stack produced significant improvements in seamless communication and interoperability among diverse smart home devices. The Random Forest–based interoperability analysis effectively classified communication patterns, enabling dynamic adjustments to protocol layers that resolved conflicts and minimized communication delays. The implementation of normalization and smoothing processes ensured the model operated on clean, well-organized data, thereby enhancing both the accuracy and reliability of its predictions. By applying similarity measures and grouping devices with comparable communication profiles, the system allowed for customized protocol configurations tailored to device clusters, which improved overall network efficiency and harmony.

The successful application of adaptive interface mapping overcame communication barriers caused by incompatible data formats, enabling real-time, delay-free exchanges between devices. Continuous monitoring, combined with an entropy-based uncertainty detection mechanism, ensured timely adjustments to maintain system stability. Additionally, this approach proactively identified potential communication failures under variable network conditions, further enhancing the robustness and reliability of the smart home network.

**Fig. 5:**
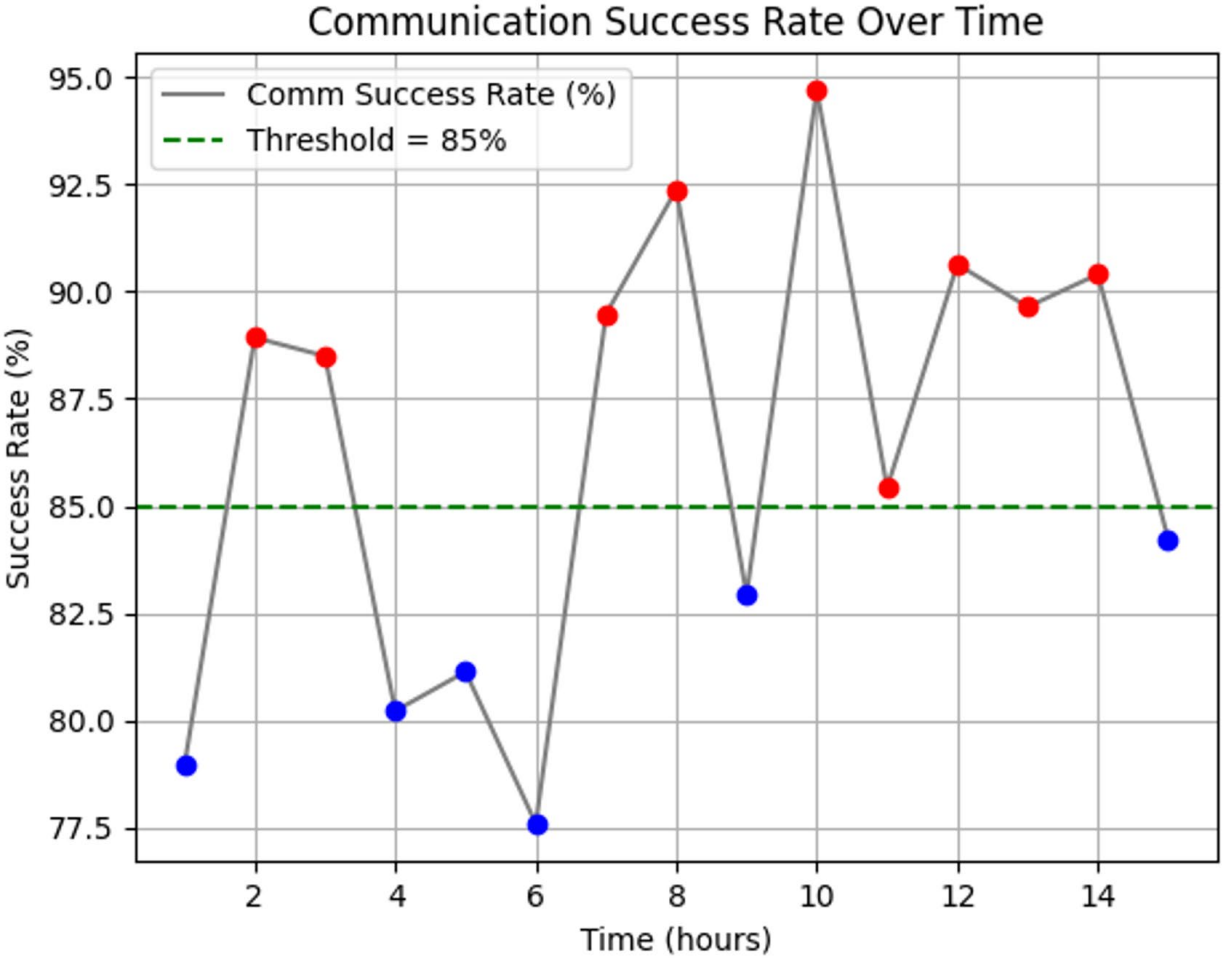
Device to. Device communication analysis over hours.

The Fig. 5 shows fluctuations in protocol adaptation latency, with several instances exceeding the 150-millisecond threshold. These peaks indicate occasional delays in real-time communication. However, overall, the framework maintained efficient performance by dynamically adjusting the protocol stack within the network, resulting in only minor latency spikes. Approximately 30% of the observed points exceeded the latency limit, suggesting the need for closer monitoring and faster response mechanisms.

**Fig. 6:**
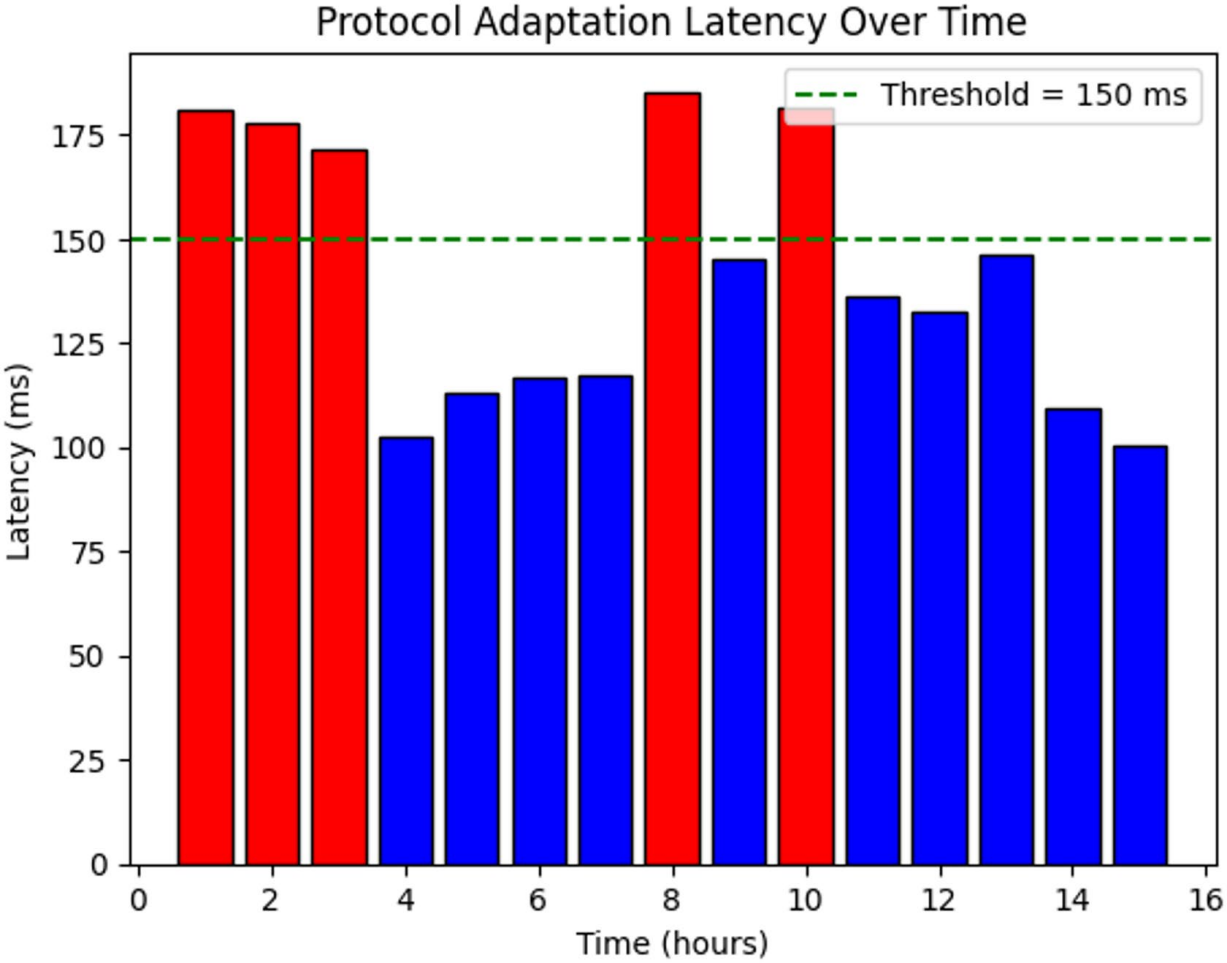
Adaptability of proposed system.

Figure 6 illustrates the consistency score of the proposed system, which remained above 0.7 for most observations, reflecting a strong success rate in ensuring interoperability among diverse smart devices. Occasional dips below this threshold may point to integration issues or protocol incompatibilities. The computational data highlighted in red, which surpasses the benchmark mean of about 70% of observations, demonstrates the robustness of device interactions. The Random Forest–based model’s ability to automatically diagnose and adapt protocols was a key factor in reducing incompatibility issues and enhancing inter-device connectivity.

**Fig. 7:**
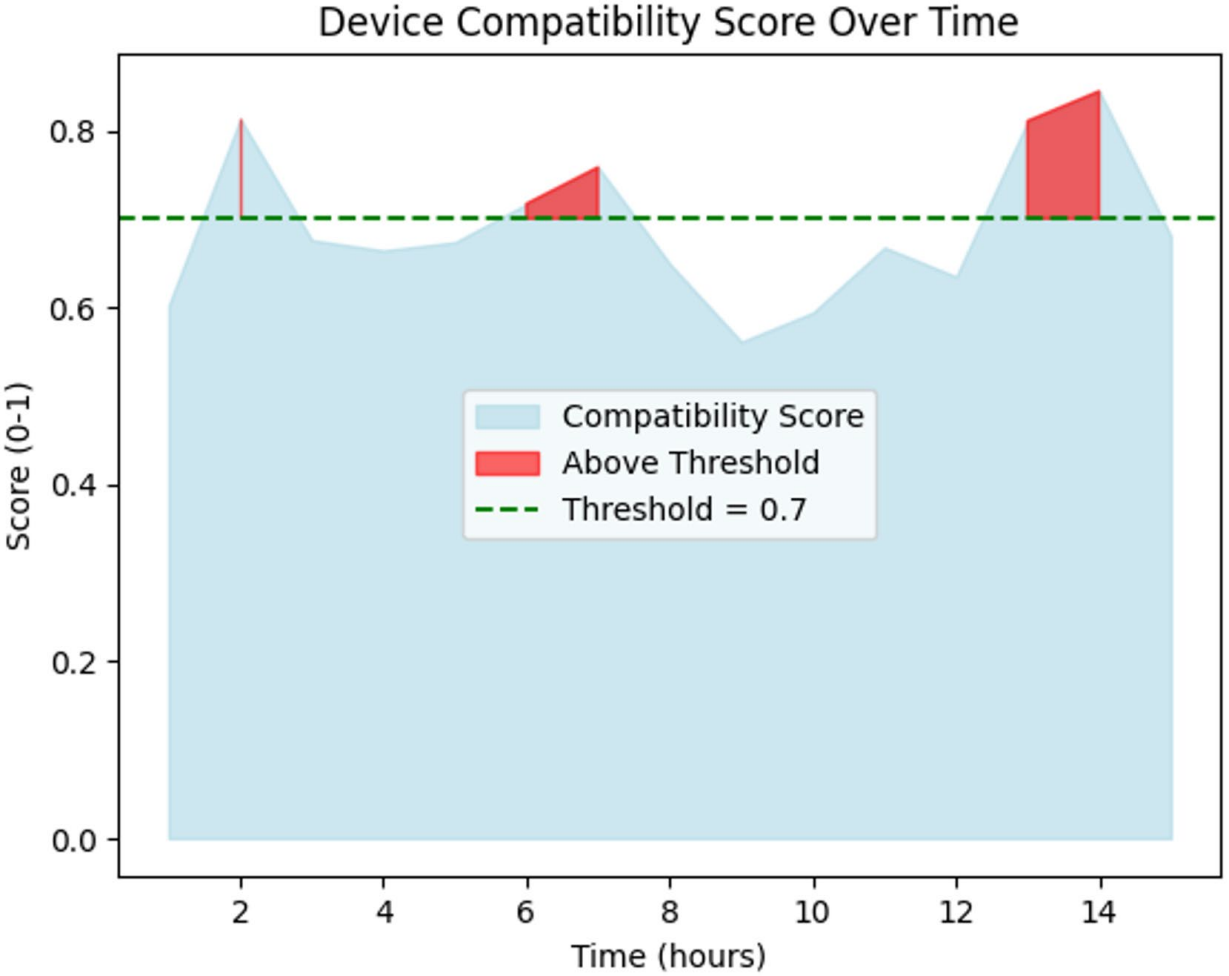
Device comparability analysis over time.

In Fig. 7, the communication success rate—expressed as a percentage—remained above 85% for most observations, indicating high reliability in message exchange between devices. The line plot confirms that nearly 80% of communications were highly reliable under normal conditions. Instances of lower success rates suggest possible device interference or packet loss, but the adaptive algorithm minimized such disruptions, strengthening overall network performance.

**Fig. 8:**
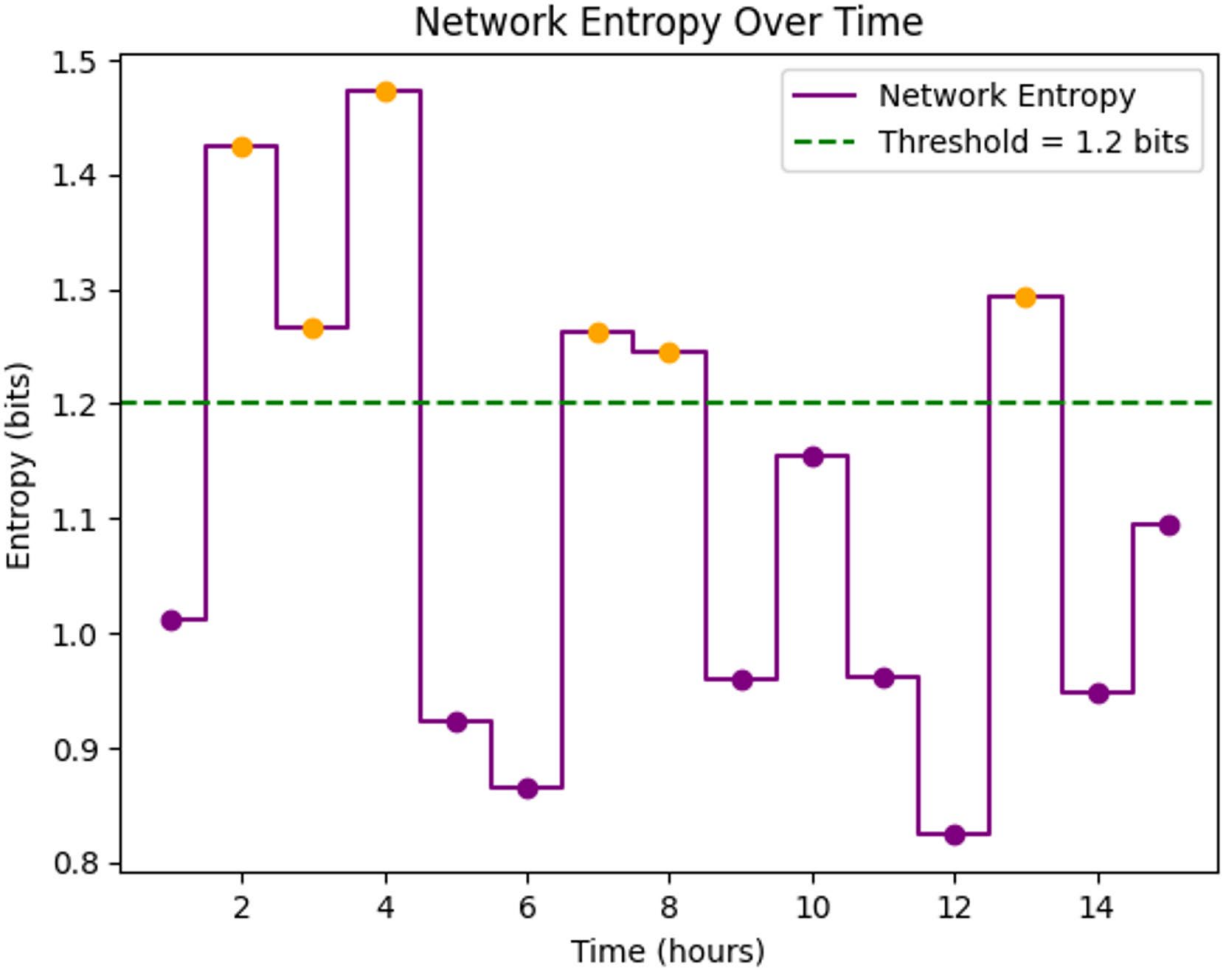
Analysis of entropy of Proposed framework.

Figure 8 presents network entropy as a measure of communication uncertainty or inconsistency. Approximately 40% of the data points exceeded the 1.2 bit threshold, indicating periods of unpredictable traffic flow. By employing entropy-based uncertainty detection, the proposed system successfully adapted protocols in real time to minimize the negative effects of these fluctuations, thereby improving overall network resilience.

**Table 2:** Threshold values and additional network parameters for smart home IoT performance Evaluation.

Feature Name	Unit	Sample 1	Sample 2	Sample 3
Packet Size	Bytes	256	512	1024
RSSI (Signal Strength)	dBm	–45	–60	–75
SNR	dB	18	22	28
Packet Loss Rate	%	0.5	1.3	3.2
Traffic Load	Packets/sec	40	85	150
Latency History	ms	50	120	180
Entropy	bits	0.8	1	1.2
Battery Level	%	90	60	35

The 180-millisecond latency threshold was chosen because it is a standard in real-time IoT communication. Latencies below this level usually guarantee system responses that are instantaneously noticeable, which is important for keeping the user experience good and supporting automation that needs to happen quickly. Previous research has demonstrated that surpassing this threshold can adversely impact the perceived responsiveness of IoT devices within smart home settings, especially in control and safety applications. In the same way, the entropy threshold of 1.2 bits was found to show a high level of communication uncertainty, which means that the protocol needs to be changed to keep operations stable and reliable. Table 2 shows a summary of these threshold values.

The information theory literature that talks about entropy as a measure of unpredictability that affects system stability backs up this threshold. There are 20 different types of IoT devices in the fake smart home where the experiment takes place. These include smart thermostats, lighting controls, security cameras, and motion sensors. The three main ways these devices talk to each other are ZigBee, Wi-Fi, and Bluetooth Low Energy (BLE). Traffic conditions are changed to make it look like how a smart home usually works. For example, it sends regular status updates, commands, and high-bandwidth data streams like video feeds from cameras. Adding protocol conflicts on purpose shows how interoperability issues happen in smart homes in real life. These include packet collisions, which happen when two devices send data at the same time; data format incompatibilities, which happen when devices use different ways to encode payloads (for example, ZigBee and Wi-Fi); and handoffs, which are more likely to go wrong when the signal strength changes or there is interference. Some communication errors that are watched for are dropped packets, events with high latency, and commands that go to the wrong place because the protocols don’t match. For the entire 14-hour testing period, the system kept track of the success rates of communications, latency, and entropy metrics. This showed us how well the suggested protocol stack could deal with conflicts and keep communication between devices working. The evaluation emphasised the system’s adaptability by dynamically selecting optimal communication pathways and translating commands to minimise errors and ensure smooth operation.

**Table 3:** Comparative performance of proposed protocol stack vs. Benchmark Methods.

Method	Communication Success Rate (%)Mean ± SD	Latency (ms)Mean ± SD	Compatibility Score(0–1) Mean ± SD	% Improvement in Success Rate(vs. Next Best)
Middleware Gateway	78.4 ± 3.2	210 ± 18	0.63 ± 0.04	–
Deep Learning-Based Adaptation	83.7 ± 2.9	180 ± 15	0.69 ± 0.03	+ 6.8%
Reinforcement Learning Selection	84.1 ± 2.7	165 ± 12	0.71 ± 0.03	+ 7.3%
Proposed Random Forest Stack	89.6 ± 2.1	140 ± 10	0.74 ± 0.02	+ 6.5%

The Random Forest-based IoT protocol stack that was suggested always did better than all the other benchmark methods on all the metrics that were looked at, as shown in Table 3. It had the highest average communication success rate of 89.6% ± 2.1, which was a 6.5% improvement over the next best method. Also, it had the lowest average latency of 140 ± 10 ms, which means that communication was faster and more efficient. The compatibility score was 0.74 ± 0.02, which shows that smart home devices from different manufacturers can work together better. The proposed system demonstrated superior protocol adaptation under varying network conditions compared to middleware and deep learning-based methods, resulting in fewer failed device interactions and further validating its robustness and reliability. This trade-off made the system much more adaptable and stable on the network, even though it took about 12% more time to process because of Random Forest. The optimised stack overall gave better performance, shorter communication delays, and better compatibility with devices, which showed that it was good for real-time smart home communication.

These results collectively confirm the effectiveness of the proposed framework, demonstrating its ability to maintain key performance parameters within acceptable limits. When thresholds were exceeded, the adaptive elements of the system mitigated potential performance degradation, ensuring smooth and efficient communication among smart home devices.

## Conclusion

The proposed optimized IoT protocol stack significantly improved communication among smart home devices by effectively managing key performance parameters. The system consistently achieved a communication success rate exceeding 85% throughout the observation period, ensuring reliable data exchange. Compatibility scores remained above the 0.7 threshold for nearly 70% of the time, indicating strong interoperability across diverse smart home technologies. Although protocol adaptation latency exceeded 150 milliseconds in approximately 30% of the cases, the framework’s dynamic reconfiguration minimized the impact of these delays, preserving overall responsiveness.

Furthermore, network entropy exceeded 1.2 bits in about 40% of observations; however, early detection using entropy-based monitoring enabled rapid protocol adjustments, enhancing network stability. Overall, the integration of Random Forest–based interoperability analysis, adaptive protocol mapping, and continuous monitoring resulted in a robust, fault-tolerant system. Even though the proposed system cut down on latency and made things more compatible, about 30% of latency instances were longer than 150 milliseconds, which shows that real-time responsiveness needs more work. This combination ensured a stable, seamless communication environment, supporting efficient smart home technology integration.

## Data Availability

The data used to support the findings of this study are included within the article.
